# Chemical Composition and In Vitro Biological Activity of the Polar and Non-Polar Fractions Obtained from the Roots of *Eleutherococcus senticosus* (Rupr. et Maxim.) Maxim

**DOI:** 10.3390/ijms26125619

**Published:** 2025-06-12

**Authors:** Jakub Gębalski, Milena Małkowska, Ewa Kiełkowska, Filip Graczyk, Sylwia Wnorowska, Iga Hołyńska-Iwan, Maciej Strzemski, Magdalena Wójciak, Daniel Załuski

**Affiliations:** 1 Department of Pharmaceutical Botany and Pharmacognosy, Ludwik Rydygier Collegium Medicum, Nicolaus Copernicus University, 85-094 Bydgoszcz, Poland; milena.malkowska@cm.umk.pl (M.M.); 315635@stud.umk.pl (E.K.); filip.graczyk@cm.umk.pl (F.G.); daniel.zaluski@cm.umk.pl (D.Z.); 2 Department of Medical Chemistry, Medical University of Lublin, 20-093 Lublin, Poland; sylwiaw211@gmail.com; 3 Department of Pathobiochemistry and Clinical Chemistry, Ludwik Rydygier Collegium Medicum, Nicolaus Copernicus University, 85-094 Bydgoszcz, Poland; igaholynska@cm.umk.pl; 4 Department of Analytical Chemistry, Medical University of Lublin, 20-093 Lublin, Poland; maciej.strzemski@umlub.pl (M.S.); magdalena.wojciak@umlub.pl (M.W.)

**Keywords:** *Eleutherococcus senticosus*, Siberian ginseng, adaptogen, ABTS, FRAP, CUPRAC, nitric oxide, superoxide anion, hydrogen peroxide, hyaluronidase, acetylcholinesterase, butyrylcholinesterase, α-amylase, tyrosinase, chemical composition, melanoma

## Abstract

*Eleutherococcus senticosus* (ES) has been used in traditional medicine for immune-boosting, stress-reducing, and endurance-enhancing properties. In this study, the chemical composition and biological activity of polar and non-polar fractions obtained from 75% methanol *E. senticosus* roots extract were evaluated. Spectrophotometric methods were used to assess the antioxidant (DPPH, ABTS, FRAP, CUPRAC, O_2_^•−^) and anti-enzymatic (hyaluronidase, acetylcholinesterase, butyrylcholinesterase, α-amylase, and tyrosinase) activities. Metabolic profiling was carried out using HPLC-DAD and UHPLC-DAD/ESI-TOF-MS. The ethyl acetate fraction (EtOAc) showed the highest antioxidant activity with IC_50_ values of 82.73 ± 0.065 µg/mL (DPPH) and 9.92 ± 0.17 µg/mL (ABTS). The EtOAc fraction also exhibited strong anti-enzymatic effects against hyaluronidase and α-amylase (125.24 ± 12.29 and 97.34 ± 9.18 µg/mL, resp.). In turn, the hexane fraction exhibited the most potent anti-AChE activity with IC_50_ equal 245.72 ± 11.82 µg/mL. The HPLC-DAD analysis revealed the presence of caffeic acid derivatives. These results suggest that the ethyl acetate fraction may have therapeutic relevance in inflammation- and metabolic-related diseases. We perceive the potential of this fraction as a rich source of compounds with an anti-inflammatory activity; however, more advanced research in in vivo model is required.

## 1. Introduction

*Eleutherococcus senticosus* (Rupr. et Maxim.) Maxim. (ES), also known as Siberian ginseng, is a plant primarily found in Asia, particularly in regions with a cold climate. Its natural habitats include areas in Siberia, China, Mongolia, and both North and South Korea. In traditional phytotherapy apart from the roots, the fruits are also used. The key compounds found in the root and rhizome are eleutherosides. They are a heterogeneous group of compounds, which includes lignans (syringaresinol, sesamin), phenolic acids (chlorogenic acid, caffeic acid), polysaccharides, coumarins (isofraxidin), and flavonoids (kaempferol, quercetin, rutin) [1,2,3,4].

ES is mainly known for its adaptogenic properties, helping the body to cope with stress, and improving overall well-being. It reduces fatigue and enhances recovery after a physical exertion. The plant stimulates the immune system, has an anti-inflammatory activity, and helps protect cells from damage caused by free radicals [5,6,7,8,9,10]. In traditional Chinese medicine, it has been used for thousands of years as a tonic to prolong life, boost energy, and treat various ailments—from spleen deficiency to insomnia. Russian folk and official phytotherapy have recognized it as a strategic adaptogen, used to enhance performance and resilience in extreme conditions [11,12,13,14].

Clinical and preclinical studies suggest that ES may improve insulin sensitivity and lower blood glucose levels, making it a promising component in the treatment of type 2 diabetes. In animal models, beneficial effects have been observed on GLUT4 transporter expression, reduction in liver steatosis, and improvement in lipid profiles, indicating enhanced glucose and lipid metabolism [15]. In the context of the cardiovascular system, *E. senticosus* supplementation in humans has been shown to lower blood pressure, reduce arterial stiffness, and improve endothelial function by increasing nitric oxide synthase (eNOS) activity. Studies in atherosclerotic mice also demonstrated a reduction in atherosclerotic lesions and improved lipid profiles. Additionally, *E. senticosus* exhibits anti-inflammatory and antioxidant properties, contributing to vascular protection [16,17,18]. Regarding neuroprotective activity, studies in animal models of Alzheimer’s and Parkinson’s diseases indicated that *E. senticosus* extract may improve memory, motor function, and reduces neuroinflammatory and oxidative neuronal damage. In mouse models, the extract inhibited inflammatory pathways, activated protective molecular mechanisms, and improved mitochondrial function [19,20,21,22].

The main objective of this study was to determine the biological properties and the phytochemical composition of fractions obtained from the methanol extract of the ES root. Despite the fact that this plant has been used for centuries in ethnomedicine as a remedy for inflammation and immunodeficiencies, the chemical composition and biological activity of this species cultivated in Poland are unknown.

Our studies on ES have revealed that its bioactivity depends on specific classes of compounds present in various extract fractions. The EtOAc fraction, rich in phenolic acids, showed strong antioxidant and inhibitory activity, suggesting that polyphenols—rather than just the traditionally studied eleutherosides—may be key to its adaptogenic effects. For the first time, the impact on the superoxide anion (O_2_^•−^) was also assessed, demonstrating the butanol fraction’s ability to scavenge it, simultaneously indicating effectiveness against biologically relevant forms of ROS. It should be mentioned that such an activity is significant in the context of oxidative stress-related diseases (such as heart disease and neurodegeneration). Strong inhibition of the digestive enzyme α-amylase was also discovered in the non-polar fraction—up to four times more potent than acarbose—suggesting antidiabetic potential and supporting the use of the plant in metabolic disorders (metabolic syndrome, obesity). In literature, there is a lack of data about interactions between ES extract and cytostatics used in chemotherapy, despite the fact that the plant is popularly used by oncology patients. Our research has revealed that ES fractions may protect cancer cells from doxorubicin toxicity, which may be risky for those patients. As can be concluded from the abovementioned statements, there is a lack of detailed research on the mechanism of ES fractions’ action; additionally, the phytochemicals and activity of ethyl acetate and butanol fractions have not yet been known. In relation to that, we have hypothesized that this species, which is cultivated in Poland, contains compounds with a desirable pharmacological activity. To prove our hypothesis, we studied four fractions in a series of in vitro models for bioactivities related to an anti-inflammatory activity.

## 2. Results and Discussion

### 2.1. Antioxidant Panel

**Figure 1:**
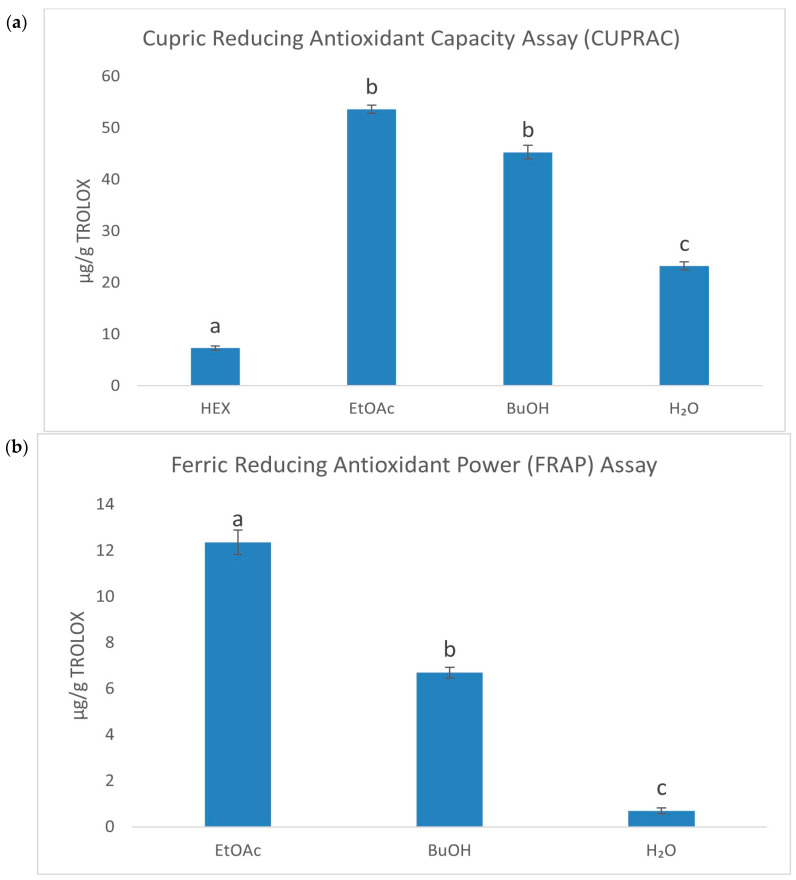

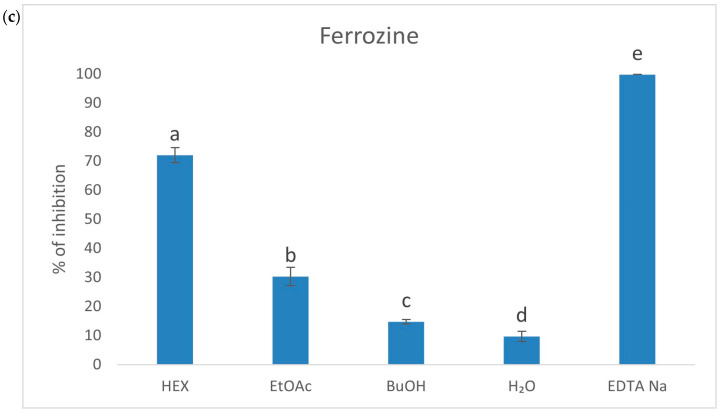
Column charts for CUPRAC (**a**), FRAP (**b**), and Ferrozine (**c**) assays were plotted at a concentration of 1 mg/mL. Different superscript lowercase letters indicate a statistically significant difference between the fractions themselves and between the fractions and control within the same column, with *p* < 0.05.

The ES root exhibited strong antioxidant properties, primarily attributed to a high content of phenolic acids, particularly chlorogenic acid. To investigate the antioxidant potential in detail, the raw extract was subjected to fractionation, resulting in four fractions with a varying polarity (HEX—hexane, EtOAc—ethyl acetate, BuOH—butanol, H_2_O—water). Based on the previous research by Gębalski et al. [23], this approach allowed for the identification of the most active fractions, with the EtOAc fraction demonstrating the strongest antioxidant activity. In order to assess the antioxidant properties of the fractions, several spectrophotometric methods were employed. Initially, the antioxidant activity of the fractions was evaluated using classic chemical methods, such as DPPH, ABTS, FRAP, CUPRAC, and iron ion chelation assays. The next step involved evaluating the fractions’ ability to scavenge reactive oxygen species (ROS). The results are presented in Table 1, Figure 1, and Supplementary Figure S1. The most active fraction against 2,2-diphenyl-1-picrylhydrazyl (DPPH), and 2,2′-azinobis-(3-ethylbenzthiazoline-6-sulfonic acid) (ABTS) radicals, was the EtOAc fraction (IC_50_ = 82.73 ± 0.065 and 9.92 ± 0.17 µg/mL, respectively). This fraction also exhibited the strongest reduction of Fe^3+^ to Fe^2+^ (12.37 ± 0.53 µg/g TROLOX) and Cu^2+^ to Cu^+^ (53.64 ± 0.79 µg/g TROLOX). The n-hexane fraction showed the strongest chelating properties (72.06 ± 2.59%). Regarding the superoxide anion radical, the butanol fraction demonstrated the strongest activity (IC_50_ = 153.54 ± 2.95 µg/mL). According to our knowledge, this is the first time when the impact of ES root’s fractions on the superoxide anion radical activity has been assessed.

Comparing our results with those reported in previous research, it can be suggested that the antioxidant activity observed in this study is strong. It is worth noting that we employed multiple tests under consistent experimental conditions, which enhances the reliability of our findings. In the study by Horng et al., ES reduced DPPH radical absorbance by 58.3 ± 2.8% at a concentration of 1000 μg/mL, indicating relatively weak free radical scavenging activity [24]. In turn, Kim et al. have evaluated the antioxidant activity of *E. senticosus* and *E. koreanum* using ABTS and DPPH tests, showing significant antioxidant properties, with IC_50_ values for ABTS of 25.4 µg/mL for *E. senticosus* and 27.8 µg/mL for *E. koreanum*, and for DPPH, 20.3 µg/mL and 22.5 µg/mL, respectively [25]. Similarly, Załuski et al. reported that the 75% ethanolic extract of ES exhibited an EC_50_ value of 0.48 mg/mL in the DPPH test, indicating its effectiveness in neutralizing DPPH radicals [26]. Our results are consistent with these findings, as the EtOAc fraction of methanolic extract exhibited higher antioxidant activity, particularly in the DPPH and ABTS tests.

Apart from the roots, the ES fruits have also been studied for their antioxidant activity. It has appeared that the EtOAc fraction exhibited strong antioxidant properties, with IC_50_ of 35.7 μg/mL for DPPH and IC_50_ of 12.1 μg/mL for ABTS [19].

When it comes to the metal ion chelation, it was found that ES demonstrated the ability to chelate Fe^2+^ ions with an EC_50_ value of 0.3 mg/mL [26]. The EtOAc fraction in our study demonstrated a chelation capacity of 63.2%, further confirming its potential role in protecting against oxidative stress. This ability was also highlighted in the FRAP test, where the EtOAc fraction reduced Fe^3+^ to Fe^2+^ with a value of 295.32 μmol Fe^2+^/g, reflecting its strong antioxidant properties [27].

Overall, our findings align with the existing literature, with the EtOAc fraction emerging as the most potent in scavenging free radicals and reducing metal ions. The comprehensive antioxidant profile of ES root extracts, especially the EtOAc fraction, underscores the potential of this plant as a promising source of natural antioxidants for protection against oxidative stress.

### 2.2. Anti-Enzymatic Panel

*Eleutherococcus senticosus*, with its diverse phytochemical profile—including saponins, lignans, phenolic acids, and coumarins—exhibits a wide array of biological activities. This section explores the effects of the fractions on various activities, including anti-inflammatory effects (via hyaluronidase inhibition), anti-neurodegenerative activity (through the inhibition of acetylcholinesterase and butyrylcholinesterase), metabolic activity (via α-amylase inhibition), and anti-aging activity (through tyrosinase inhibition). The results are presented in Table 2 and Supplementary Figure S2. The most active fraction against hyaluronidase was the EtOAc fraction (IC_50_ = 125.24 ± 12.29 μg/mL), which showed higher activity than the positive control, escin (IC_50_ = 390.8 ± 0.81 μg/mL). In previous studies, the EtOAc fraction from the root of *Eleutherococcus divaricatus* (ED) exhibited the strongest activity, with an IC_50_ value of 27.50 ± 0.65 μg/mL. In the case of hyaluronidase from the serum of children with acute lymphoblastic leukemia, the average inhibitory activity of the EtOAc fraction was 55.82% at a concentration of approximately 34 µg/mL [23]. In the case of the 75% methanol extract from ED fruits, the IC_50_ against bovine hyaluronidase was 450 ± 40 µg/mL, while for human hyaluronidase, the inhibition value at a concentration of approximately 34 µg/mL ranged from 76 to 86% [28].

Also, the fractions of ES showed a potential antidiabetic activity. Both the n-hexane fraction and the ethyl acetate fraction inhibited the enzyme stronger than acarbose (IC_50_ = 97.34 ± 9.18, 281.63 ± 7.77, and 384.34 ± 27.84 μg/mL, resp.). Similar results were obtained for the methanol extract from the leaves of *E. gracilistylus*, which inhibited α-amylase (IC_50_ = 30.81 ± 1.04 μM, acarbose—854.43 ± 0.81 μM) and α-glucosidase activity (IC_50_ = 13.01 ± 0.38 μM, acarbose—661.73 ± 0.48 μM) [29]. In the study by Lim et al., the impact of 80% ethanol extracts from the leaves of *E. senticosus*, *E. gracilistylus*, *E. sieboldianus*, and *E. sessiliflorus* on the activity of amylase and α-glucosidase was investigated. For amylase, the IC_50_ values were 7.24, 25.15, 68.73, and 99.44 μg/mL for extracts, and for acarbose it was 5.45 mg/mL. In the case of α-glucosidase, the inhibition for the extract at concentration of 10 mg/mL was 35.94 ± 2.81%, 37.81 ± 2.45%, 21.09 ± 2.39%, and 23.58 ± 3.39%, resp., and 87.15 ± 1.01% for acarbose [30].

Regarding anti-tyrosinase activity of fractions, the most active fraction was the ethyl acetate, with an IC_50_ of 147.12 ± 8.28 μg/mL, compared to kojic acid, used as a control, with an IC_50_ of 34.33 ± 1.22 μg/mL. There is little information in the literature about the effect of the *Eleutherococcus* genus on tyrosinase. In previous studies, the acetate, butanol, and water fractions of the *E. divaricatus* root extract exhibited significant inhibitory properties against fungal tyrosinase, with IC_50_ values of 65.50 ± 1.35, 85.40 ± 2.51, and 81.10 ± 5.32 μg/mL, respectively. The methanolic extract from *E. divaricatus* fruits showed a weak inhibition, with an IC_50_ value of 2670 μg/mL. In both cases, the extracts and fractions did not affect the human enzymes in the serum of children with acute lymphoblastic leukemia [23,28].

In the next part, the potential anti-dementia activity of the fractions, expressed as AChE and BuChE inhibition, was examined. The hexane fraction exhibited the strongest inhibitory properties against AChE and BuChE, with IC_50_ values of 280.00 ± 49.72 and 245.72 ± 11.82 μg/mL, respectively. In the study by Załuski et al., the effects of 75% EtOH and chloroform extracts from the roots of *E. setchuenensis*, *E. divaricatus*, *E. henryi*, *E. sessiliflorus*, and *E. gracilistylus* on AChE and BuChE activity were tested. In the case of the ethanol extract, *E. setchuenensis* and *E. sessiliflorus* were the most active against AChE (for both IC_50_ = 0.3 ± 0.06). For BuChE, *E. henryi* showed the highest activity (IC_50_ = 0.13 ± 0.02 mg/mL). Regarding the chloroform extracts, the most active extract against both AChE and BuChE was from *E. gracilistylus* (IC_50_ = 0.37 ± 0.05 and 0.12 ± 0.04 mg/mL). For *E. setchuenensis*, the ethanol extract had anti-AChE and anti-BuChE activities with the IC_50_ = 0.46 ± 0.04 and 0.73 ± 0.1 mg/mL, respectively, while for the chloroform extract, the IC_50_ values were 0.75 ± 0.08 and 0.71 ± 0.08 mg/mL [31]. In another study, the effect of 75% methanol extracts from the roots of *E. senticosus*, *E. divaricatus*, *E. sessiliflorus*, *E. gracilistylus*, and *E. henryi* on AChE was examined. The most active were *E. gracilistylus* and *E. sessiliflorus*, with the inhibition at a concentration of 1 mg/mL of 32 ± 0.8% and 32 ± 0.6%, respectively. For *E. senticosus*, the inhibition was equal to 26.1 ± 0.05% [32]. In another study, methanolic, dichloromethane, and aqueous extracts made from ES root did not show any activity against AChE [33].

The obtained results highlight the broad therapeutic potential of the *E. senticosus* roots. The inhibition of hyaluronidase activity, along with significant antioxidant properties, support its use in the treatment of inflammatory diseases. Its activity against amylase substantiates its role as an adjunct in the management of diabetes and obesity.

### 2.3. Chemical Panel

Plant extracts constitute a complex mixture of chemical compounds with a varied polarity. To gain a more detailed understanding of the composition of the methanolic extract from the ES root, it was subjected to extraction using solvents of varying polarity. The largest mass of fraction was obtained for the BuOH, equal to 9.89 g. The obtained fractions were then thoroughly analyzed using spectrophotometric and chromatographic methods. Initially, simple chemical reactions were used to determine the total content of polyphenols, flavonoids, and phenolic acids. The results are presented in Table 3. The EtOAc fraction was the richest in polyphenols (26.77 ± 1.10 mgGAE/g), flavonoids (21.35 ± 3.34 mgQE/g), and phenolic acids (3.13 ± 0.54 mgCAE/g). Literature provides limited information on the polyphenolic content in *Eleutherococcus* species, particularly concerning the fractions. Adamczyk et al. reported that the polyphenol and flavonoid contents in 75% MeOH extracts were equal to 7.9 ± 0.3 gGAE/g and 4.6 ± 0.9 gQE/g, respectively [32]. The concentrations of polyphenols, flavonoids, and phenolic acids in the hydrophobic–hydrophilic extract from the roots of *E. senticosus* enriched with naringenin were 159.27 ± 2.73 mg GAE/g, 137.47 ± 5.23 mg QE/g, and 79.99 ± 3.57 mg CAE/g, respectively [34].

Biological activity is linked with the presence of secondary metabolites; therefore, the quantitative HPLC-DAD analysis was performed to evaluate the content of phenolic acids and eleutheroside B (Table 4). The analyzed compounds included protocatechuic acid (PA), caffeic acid (CA), chlorogenic acid (ChA), 4,5-dicaffeoylquinic acid (4,5-DCA), 3,5-dicaffeoylquinic acid (3,5-DCA), and eleutheroside B (Eleuth B). Among the tested solvents, ethyl acetate demonstrated the highest extraction efficiency for most compounds. It yielded the highest content of caffeic acid (9.96 ± 0.05 mg/g), chlorogenic acid (117.57 ± 0.87 mg/g), 4,5-dicaffeoylquinic acid (16.58 ± 0.71 mg/g), and 3,5-dicaffeoylquinic acid (103.36 ± 0.39 mg/g). These results indicate that ethyl acetate is the most effective solvent for extracting phenolic acids and dicaffeoylquinic acids.

**Figure 2:**
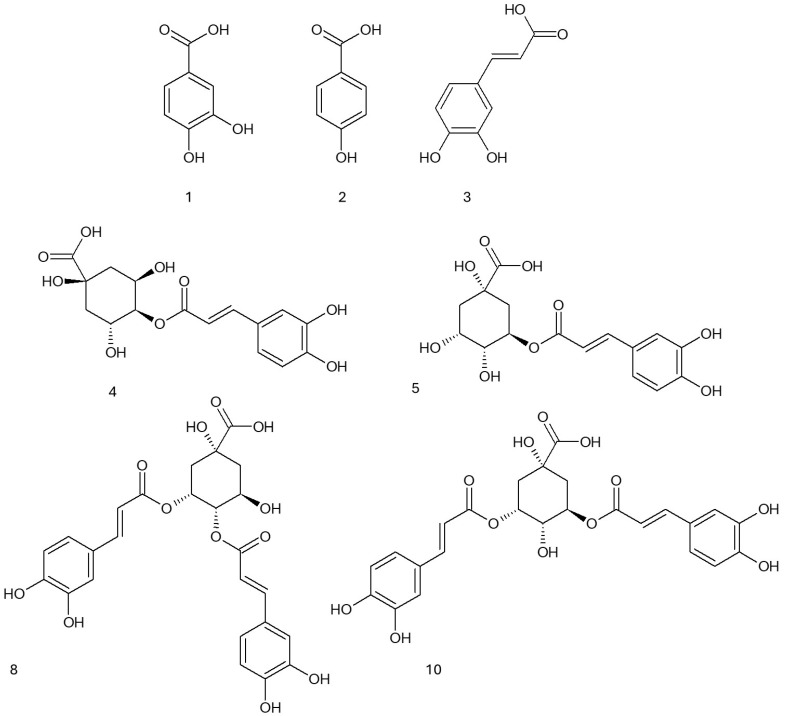
Formulae of the compounds present in the EtOAc fraction according to Table 5.

In the next step, the most active fraction, the ethyl acetate, was analyzed by means of UHPLC-DAD/ESI-TOF-MS, allowing for an identification of compounds belonging to three main groups such as benzoic acid derivatives, cinnamic acid derivatives, and depsides. The results are shown in Table 5 and illustrated in Figure 2.

The benzoic acid derivatives included protocatechuic acid (7.68 ± 0.18 mg/g) and hydroxybenzoic acid, detected in trace amounts. The cinnamic acid derivatives comprised caffeic acid (8.64 ± 0.31 mg/g), *p*-coumaroylquinic acid (1.78 ± 0.10 mg/g), and feruloylquinic acid (2.61 ± 0.20 mg/g).

The largest group of identified compounds were depsides, with chlorogenic acid as the predominant representative (118.5 ± 6.21 mg/g). Additionally, its isomer, cryptochlorogenic acid (6.81 ± 1.0 mg/g), was also detected along with several dicaffeoylquinic acid derivatives, including 3,5-dicaffeoylquinic acid (75.1 ± 4.2 mg/g), dicaffeoylquinic acid (93.3 ± 3.30 mg/g), and 4,5-dicaffeoylquinic acid (7.11 ± 0.23 mg/g).

The high content of depsides and caffeic acid derivatives may indicate their significant impact on the biological properties of the analyzed material. In our previous studies, the ethyl acetate fraction obtained from the methanolic extract of *Eleutherococcus divaricatus* mainly contained derivatives of cinnamic acid (chlorogenic acid, caffeic acid, 3,5-dicaffeoylquinic acid, 4,5-dicaffeoylquinic acid) and benzoic acid (protocatechuic acid, hydroxybenzoic acid) [23]. In the study by Jin et al., HPLC-DAD-MS analysis of the ethanolic extracts from the bark and root of ES revealed the high content of syringine, caffeic, and isoferulic acids in the root extract in a comparison to the bark extract. On the other hand, the bark extract showed higher concentrations of sesamine and oleanolic acid [35]. In the study by Bączek et al., the methanolic extract from the roots and rhizomes of ES contained lignans (eleutheroside E and eleutheroside E1), eleutheroside B, phenolic acids (chlorogenic acid, rosmarinic acid, protocatechuic acid, caffeic acid), and sterols (sitosterol 3-O-*β*-D-glucoside—eleutheroside A, *β*-sitosterol, campesterol, brassicasterol) [36]. A comparable phytochemical profile was observed in methanolic extracts from ES root, which were rich in caffeoylquinic acid derivatives. These included 5-O-caffeoylquinic acid and three isomeric compounds, 1,5-O-dicaffeoylquinic acid, 3,5-O-dicaffeoylquinic acid, and 4,5-O-dicaffeoylquinic acid [37].

### 2.4. Cytotoxicity Panel

**Figure 3:**
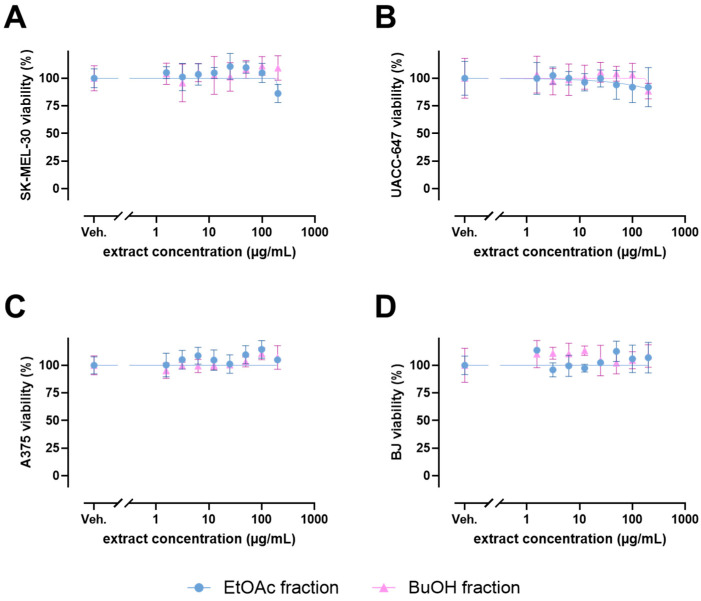
Viability of SK-MEL-30 (**A**), UACC-647 (**B**), A375 (**C**), and BJ (**D**) cell lines plotted against extract concentrations on logarithmic x-axes. The data are presented as mean ± SD and originate from three independent biological experiments.

To evaluate the general biological impact of ES extract fractions, three different human melanoma cell lines were selected: SK-MEL-39, UACC-647, and A375, accompanied by normal BJ fibroblasts. The cells were exposed to the increasing concentrations (up to 200 µg/mL) of either EtOAc or BuOH fraction (Figure 3). No significant alterations in cell viability across the tested cell lines were observed, indicating that the fractions are not toxic to cells of skin origin.

**Figure 4:**
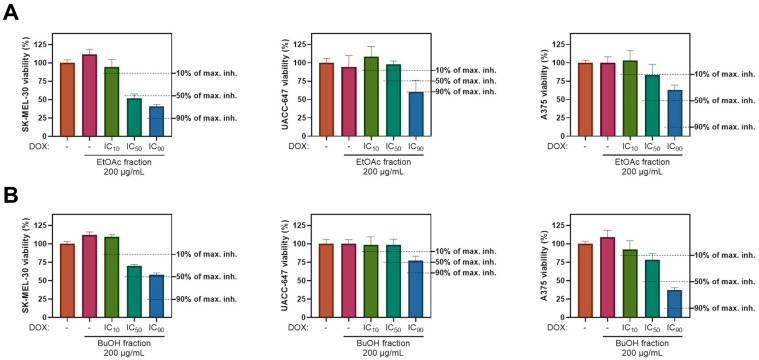
Effect of co-treatment with doxorubicin and *E. senticosus* fractions of the viability of SK-MEL-30, UACC-647, and A375 melanoma cells. The cells were exposed to IC_10_, IC_50_, or IC_90_ of doxorubicin (DOX) and with either EtOAc fraction (**A**) or BuOH fraction (**B**) at the dose of 200 µg/mL. Levels of 10%, 50%, and 90% of maximal inhibition elicited by DOX were marked as horizontal dotted lines.

Compounds of natural origin are frequently used in parallel to chemotherapy as part of complementary treatments. Thus, we investigated the impact of the ES extract on cell viability in the presence of the chemotherapeutic agent doxorubicin (DOX). Melanoma cell lines were treated with 200 µg/mL of the EtOAc or BuOH fractions simultaneously with DOX at concentrations corresponding to its IC_10_, IC_50_, or IC_90_ values. These DOX concentrations were previously determined for each cell line individually [23]. Both fractions elicited a protective effect against the DOX-induced toxicity in all three melanoma cell lines (Figure 4). For instance, A375 cells subjected to the EtOAc fraction and IC_90_ of DOX experienced approximately a 37% drop in viability (Figure 4A, third panel), whereas DOX alone inhibited the viability of A375 cells by 96.38% (at IC_90_).

In a previous study, the ethyl acetate fraction of *E. divaricatus* did not affect the viability of normal fibroblasts or melanoma cell lines (SK-MEL-30, UACC-647, A375) at doses up to 200 µg/mL, suggesting no cytotoxicity. When combined with DOX, the EtOAc fraction of *E. divaricatus* reduced DOX-induced cytotoxicity, offering a protective effect, especially in A375 cells [23]. Similar results were obtained in a study on a preparation containing a hydrophobic-hydrolysable extract from the root of ES and naringenin, as well as an extract from ES fruits, in the inhibition of the FaDu cell line (malignant pharyngeal carcinoma) and HepG2 cells (human liver cancer cell line). The results did not reveal cytotoxic effects of these substances; on the contrary, a slight increase in cell survival was recorded, supporting the hypothesis that adaptogens, such as ES, rather do not harm cells, including cancer cells [33]. Similar results were obtained for another cancer cell line, HL-60, which was stimulated with 75% methanolic extracts from the roots of *E. senticosus*, *E. divaricatus*, *E. sessiliflorus*, *E. gracilistylus*, and *E. henryi*. The strongest cytotoxic activity, measured by the inhibition of HL-60 cell growth, was demonstrated by the *E. henryi* extract, achieving an IC_50_ value of 270 μg/mL [32].

### 2.5. Statistical Analysis

#### 2.5.1. Principal Component Analysis

**Figure 5:**
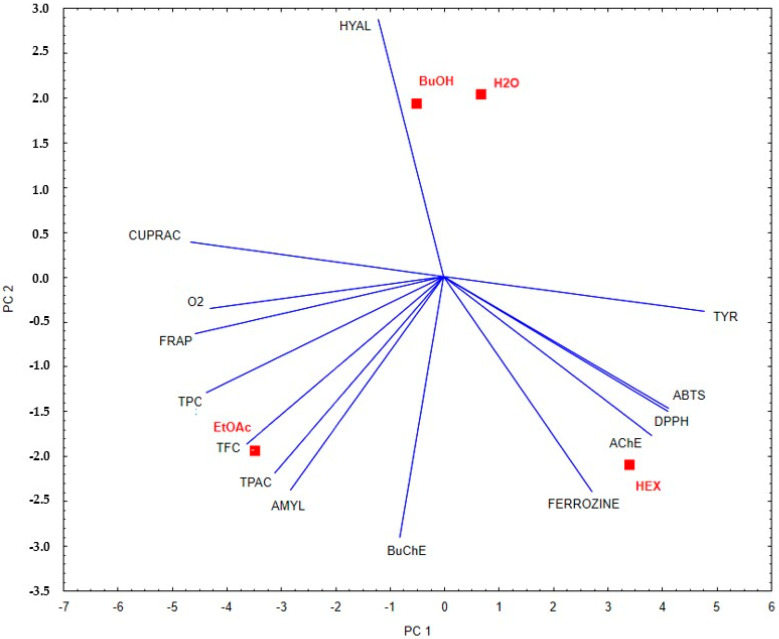
Principal component analysis of the studied fractions of *E. senticosus* based on phenolic compound content and anti-enzymatic and antioxidant activities.

Based on the PCA analysis, distinct differences in the biological activity and chemical composition of the examined plant fractions can be observed. The ethyl acetate extract (EtOAc) shows a strong correlation with vectors corresponding to the total phenolic content (TPC), total flavonoid content (TFC), and antioxidant activity as determined by FRAP and CUPRAC assays. This suggests that this fraction is characterized by a high concentration of phenolic compounds and strong antioxidant potential. In contrast, the water (H_2_O) and butanol (BuOH) extracts, located closer to the center of the PCA coordinate system, exhibit weaker yet relatively balanced correlations with the analyzed parameters. This may indicate moderate biological activity across a broad spectrum, without a clear dominance of any specific mechanism of action. The hexane extract (HEX) correlates with vectors associated with acetylcholinesterase (AChE), butyrylcholinesterase (BuChE), and hyaluronidase (HYAL) inhibition, which points to its potential neuroprotective properties and ability to inhibit enzymes involved in the progression of neurodegenerative diseases. The results are shown in Figure 5.

#### 2.5.2. Correlation Analysis

The analysis of the correlations between the total phenolic content (TPC), flavonoid content (TFC), and phenolic acid content (TPAC) with antioxidant and enzymatic activity has revealed several key relationships.

In terms of antioxidant activity, FRAP and CUPRAC show a strong positive correlation with TPC, TFC, and TPAC. This indicates that the higher the polyphenols content, the greater the ability to reduce metal ions, suggesting an antioxidant mechanism based on electron transfer. Ferrozine, which measures iron chelation capacity, shows a very weak correlation with TPC, TFC, and TPAC, suggesting that phenolic compounds in ES do not strongly influence iron chelation. Interestingly, the superoxide radical (O_2_^•−^) displays a moderate positive correlation with TFC and TPAC, implying that flavonoids and phenolic acids may play a more significant role.

**Figure 6:**
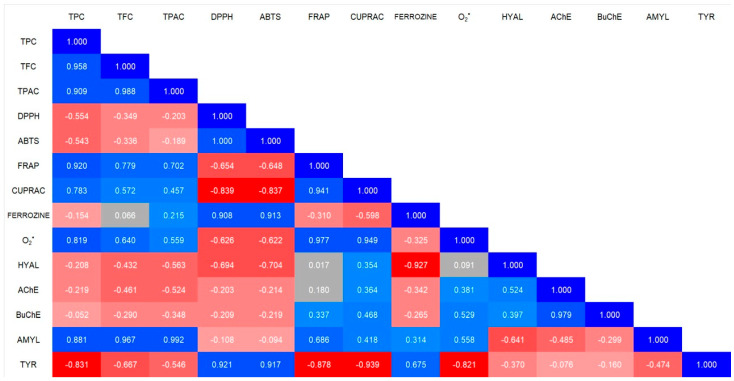
Correlation analysis (R) between the antioxidant activity, anti-enzymatic activity, and chemical composition of *E. senticosus* root. The color scale encodes Pearson correlation coefficients: progressively deeper blues denote stronger positive correlations, whereas increasingly saturated reds indicate stronger negative correlations.

Regarding enzymatic activity, amylase (AMYL) exhibits a strong correlation with TPC, TFC, and TPAC, suggesting that phenolic compounds may significantly influence carbohydrate-digesting enzymes. In contrast, acetylcholinesterase (AChE) and butyrylcholinesterase (BuChE) do not exhibit strong correlations with any of the studied parameters, indicating that polyphenols may not have a significant impact on these neuroprotective enzymes. The data are presented in Figure 6.

## 3. Materials and Methods

### 3.1. Chemicals and Reagents

The following standards and reagents were obtained from Sigma-Aldrich (St. Louis, MO, USA): eleutheroside B (≥98.0% HPLC), eleutheroside E (≥98.0% HPLC), protocatechuic acid (≥97%), *p*-hydroxybenzoic acid (99%), vanillic acid (≥97%), caffeic acid (≥98%), ferulic acid (≥99%), rosmarinic acid (≥98%), ascorbic acid, 2(3)-t-butylhydroquinone monomethyl ether (BHA), 2(3)-t-butyl-4-hydroxyanisole, hyaluronic acid (IV), aescin (>95%), hyaluronidase from bovine testes, L-tyrosine (≥98%), kojic acid, and mushroom tyrosinase. Additionally, the following compounds were sourced from Sigma-Aldrich: 2,2-diphenyl-1-picrylhydrazyl (DPPH), 2,2′-azinobis-(3-ethylbenzthiazoline-6-sulfonic acid) (ABTS), potassium persulfate, 3-(2-Pyridyl)-5,6-diphenyl-1,2,4-triazine-p,p′-disulfonic acid monosodium salt hydrate (ferrozine), iron(II) chloride tetrahydrate (>98%, FeCl_2_ × 4H_2_O), 1,3,5-Tri(2-pyridyl)-2,4,6-triazine (TPTZ), iron (III) chloride (≥98%, FeCl_3_), aluminum chloride (AlCl_3_), potassium acetate, Folin–Ciocalteu reagent, sodium nitrite, sodium molybdate, and 6-hydroxy-2,5,7,8-tetramethylchroman-2-carboxylic acid (Trolox), sodium carbonate (Na_2_CO_3_), xanthine solution, nitroblue tetrazolium chloride (NBT), xanthine oxidase, acetate buffer, bovine serum albumin (BSA), sodium chloride (NaCl), cetyltrimethylammonium bromide (CTAB), acetylcholine, 5,5′-dithiobis-(2-nitrobenzoic acid) (DNTB), donepezil, acarbose, iodine solution, phosphate buffer, copper (II) chloride (CuCl_2_), and neocuproine. The following solvents, used for extraction, were sourced from Avantor Performance Materials (Gliwice, Poland): gradient-grade acetonitrile, trifluoroacetic acid (≥99%), phosphate-buffered saline (PBS), DMEM, and RPMI 1649 Medium, among others.

### 3.2. Preparation of Raw Material, Extraction, and Isolation

The roots of ES were collected in October 2020 from the Arboretum of the Warsaw University of Life Sciences in Rogów, Poland. The identification of the raw material was performed by Prof. Daniel Załuski.

To prepare the 75% methanolic extract of ES, 94.304 g of dried root powder was extracted with 1 L of 75% methanol solution over three days at room temperature. The extraction flask was placed in an ultrasonic bath for 15 min to enhance the process. Afterward, the mixture was filtered, and the extract was concentrated under reduced pressure at 40 °C, after which 100 mL of hexane was added to the residue. The extraction was repeated three times, and the collected hexane fractions were combined. The remaining residue was evaporated and dissolved in 10% methanol. The resulting mixture was extracted three times with 100 mL of ethyl acetate (EtOAc), and the combined EtOAc fractions were collected. The remaining aqueous phase was extracted in a similar manner with n-butanol (n-BuOH). The resulting fractions were concentrated under reduced pressure at 40 °C and stored at –20 °C until further analysis.

### 3.3. Antioxidant Panel

#### 3.3.1. DPPH Free Radical Scavenging Activity

The assay was performed in a 96-well plate format [38]. Briefly, the working DPPH solution was prepared by dissolving 24 mg of DPPH in 100 mL of methanol. The DPPH solution was adjusted with methanol until an absorbance of 0.900 ± 0.03 was achieved at 515 nm. Five concentrations of each fraction were tested: 1–0.001 mg/mL. Briefly, 10 μL of the extract was mixed with 190 μL of DPPH solution. The background control consisted of 10 μL of 100% DMSO and 190 μL of DPPH solution. Ascorbic acid, BHA, and Trolox were used as reference antioxidants. The plate was incubated in the dark for 30 min, and absorbance was measured at 515 nm. The percentage of DPPH radical scavenging activity was calculated using the following formula:

$${INH}_{DPPH}=(\frac{A_{C}-A_{S}}{A_{C}})\times 100\%$$

A_S_—absorbance of the sample + DPPH

A_C_—absorbance of the DPPH

The IC_50_ values were calculated using the following formula:

$$Y=\frac{A_{1}{-A}_{2}}{1-({\frac{X}{{\mathrm{IC}}_{50}})}^{p}}+A_{2}$$

Y = % scavenging activity

X = concentration (µg/mL)

A_1_, A_2_ = maximum and minimum asymptote values (top and bottom of the curve)

p = slope factor

IC_50_ = the concentration at which 50% of the maximum scavenging activity is observed

#### 3.3.2. ABTS Free Radical Scavenging Activity

This study was conducted on a 96-well plate [39]. To generate the ABTS solution, 10 mL of a 7 mM ABTS aqueous solution was mixed with 10 mL of a 2.45 mM potassium persulfate solution. The mixture was then left to incubate in the dark for 12 h. After incubation, the solution was diluted with water until its absorbance at 405 nm reached 0.700 ± 0.03. Five concentrations of each fraction were used in the study: 1–0.001 mg/mL. Briefly, 10 μL of fractions was mixed with 190 μL of ABTS solution in potassium persulfate. The background was 10 μL of 100% DMSO, 190 μL of ABTS. Ascorbic acid, BHA, and Trolox were used as standards. The plate was incubated in a dark place for 30 min, and the absorbance was measured at 405 nm. The following formula was used to convert the obtained data:

$${INH}_{ABTS}=(\frac{A_{C}-A_{S}}{A_{C}})\times 100\%$$

A_S_—absorbance of the sample + ABTS

A_C_—absorbance of the ABTS

The IC_50_ values were calculated using the following formula:

$$Y=\frac{A_{1}{-A}_{2}}{1-({\frac{X}{{\mathrm{IC}}_{50}})}^{p}}+A_{2}$$

Y = % scavenging activity

X = concentration (µg/mL)

A_1_, A_2_ = maximum and minimum asymptote values (top and bottom of the curve)

p = slope factor

IC_50_ = the concentration at which 50% of the maximum scavenging activity is observed

#### 3.3.3. Iron (II) Ion Chelation Assay

This study was conducted on a 96-well plate [40]. A concentration of each fraction was used in the study: 1 mg/mL. An amount of 140 μL of methanol, 5 μL of FeCl_2_ solution, and 100 μL of extract were added to the well. Control samples (140 μL of methanol, 5 μL of FeCl_2_ solution, and 100 μL of 100% DMSO) were also performed. The plate was incubated for 5 min at 25 °C, and 5 μL of ferrozine solution was added to each well. The plate was then incubated for 10 min at 25 °C, and the absorbance was measured at 517 nm. The following formula was used to convert the obtained data:

$${INH}_{Chel.}=(\frac{A_{C}-A_{S}}{A_{C}})\times 100\%$$

A_S_—absorbance of the sample + Ferrozine + Fe^2+^

A_C_—absorbance of the Ferrozine + Fe^2+^

#### 3.3.4. O_2_^•–^ Scavenging Capacity Assay

The scavenging activity of the superoxide anion was assessed using the xanthine–xanthine oxidase system, with nitro blue tetrazolium chloride (NBT), as described by Choi et al. [41]. Briefly, 50 μL of fraction at concentrations of 10–0.01 mg/mL (equivalent to 500, 50, 5, 0.5 μg/200 μL, respectively), 100 μL of a xanthine solution (0.4 mM) and NBT (0.24 mM) in a 1:1 (*v*/*v*) ratio, and 50 μL of xanthine oxidase (10 mU) were combined. The mixture was incubated for 20 min at 37 °C, and absorbance was measured at 560 nm. All reagents were prepared in PBS. As a positive control, ascorbic acid was used.

$${INH}_{{O_{2^{\cdot }}}^{-}}=(\frac{A_{C}-A_{S}}{A_{C}})\times 100\%$$

A_S_—absorbance of the sample + NBT + xanthine–xanthine oxidase system

A_C_—absorbance of the NBT + xanthine–xanthine oxidase system

The IC_50_ values were calculated using the following formula:

$$Y=\frac{A_{1}{-A}_{2}}{1-({\frac{X}{{\mathrm{IC}}_{50}})}^{p}}+A_{2}$$

Y = % scavenging activity

X = concentration (µg/mL)

A_1_, A_2_ = maximum and minimum asymptote values (top and bottom of the curve)

p = slope factor

IC_50_ = the concentration at which 50% of the maximum scavenging activity is observed

#### 3.3.5. Ferric Ion Reducing Antioxidant Power Assay

The FRAP assay was performed by combining extracts at concentration of 1.0 mg/mL with 290 μL of a working solution containing acetate buffer (15 mL), TPTZ solution (1.5 mL), and FeCl_3_·4H_2_O (1.5 mL). The mixture was incubated for 30 min before measuring the absorbance at 593 nm. Trolox and BHA were used as positive controls. The results of the FRAP assay were expressed as microgram of Trolox per gram of the sample (µg Trolox/g sample), with the calibration curve shown in Supplementary Figure S3 [42].

#### 3.3.6. Cupric Ion Reducing Antioxidant Capacity Assay

The CUPRAC assay was performed by combining extracts at concentration of 1.0 mg/mL (10 μL) with 190 μL of a working solution containing acetate buffer (pH = 7), neocuproine solution (7.5 mM), and CuCl_2_ (10 mM) in a 1:1:1 ratio. The mixture was incubated for 15 min before measuring the absorbance at 450 nm. The results of the CUPRAC assay were expressed as microgram of Trolox per gram of the sample (µg Trolox/g sample) with the calibration curve shown in Supplementary Figure S4 [43].

### 3.4. Anti-Enzymatic Panel

All samples were dissolved in 5% DMSO. In the study, they were used at concentration of 0.01–1.0 mg/mL, respectively. The IC_50_ values were determined using linear regression (y = ax + b), where y is the percentage inhibition and x is the extract concentration.

#### 3.4.1. Hyaluronidase Inhibitory Assay

Hyaluronidase inhibitor assays were performed in 96-well plates according to a modified method described by Di Ferrante [44] and Studzińska-Sroka [45]. The activity of the compounds/extracts was determined by precipitation of the undigested hyaluronic acid with cetyltrimethylammonium bromide (CTAB). An amount of 10 μL of sample (0.45 mg/mL), 15 μL of acetate buffer (pH = 5.35), 25 μL of incubation buffer (pH = 5.35, 0.1 mg/mL BSA, 4.5 mg/mL NaCl) and 25 μL of enzyme (30 U/mL, incubation buffer) were mixed. After 10 min incubation at 37 °C, 25 μL (0.3 mg/mL in acetate buffer pH = 5.35) of a hyaluronic acid solution was added. Afterward, plates were incubated for 45 min at 37 °C. After incubation, undigested HA was precipitated by adding of 200 μL of 2.5% CTAB. The plates were kept at 25 °C for 10 min. The intensity of complex formation was measured at 600 nm. To determine the presence of inhibition, the absorbance of solution without inhibitor (AC) and enzyme (AT) were measured. All samples were tested in triplicate. Escin was used as a standard. The inhibition was calculated using the following equation:

$${INH}_{hyal}=\left(\frac{A_{S}-A_{C}}{A_{T}-A_{C}}\right)\times 100\%$$

A_S_—absorbance of the substrate + sample + enzyme

A_C_—absorbance of the substrate + enzyme

A_T_—absorbance of the substrate + sample

#### 3.4.2. Cholinesterase Inhibitory Assay

The acetylcholinesterase (AChE) and butyrylcholinesterase (BuChE) activities were measured using a modified Ellman’s assay [46]. In brief, 5 μL of the extract was incubated for 15 min with 45 μL of AChE/BuChE enzyme (0.4 U). After incubation, 150 μL of a mixture consisting of buffer, acetylcholine, and DNTB in a 308:2:1 ratio was added, and activity was measured at time 0 and after 10 min at a wavelength of 405 nm. Donepezil was used as a control. Inhibition was calculated using the following formula:

$${INH}_{AChE/BuChE}=\left(1-\frac{As}{At}\right)\times 100\%$$

A_S_—absorbance of the substrate + sample + enzyme

A_C_—absorbance of the substrate + enzyme

#### 3.4.3. α-Amylase Inhibitory Assay

The method based on the staining of undigested starch by I_2_ was used to measure amylase activity [47]. In brief, 25 μL of the extract or standard (acarbose) was incubated with 50 μL of enzyme (10 U/mL) and 50 μL of starch (0.05%). After 10 min of incubation at 37 °C, 25 μL of HCl (1 M) and 100 μL of iodine solution (3%) were added. Absorbance was measured at a wavelength of 600 nm. Inhibition was calculated using the following formula:

$${INH}_{Amylase}=(1-\frac{A_{C}-A_{S}}{A_{C}})\times 100\%$$

A_S_—absorbance of the substrate + sample + enzyme

A_C_—absorbance of the substrate + enzyme

#### 3.4.4. Tyrosinase Inhibitory Assay

Tyrosinase inhibitor assays were performed in 96-well plates according to a modified method described by Gębalski et al. [48]. Briefly, 10 μL of the sample, 140 μL of phosphoric buffer (pH = 6.8), and 25 μL of an enzyme (125 U/mL in phosphoric buffer pH = 6.8) were mixed and incubated for 10 min at room temperature. In addition, a control without inhibitor was prepared (Ac). After incubation, to each well, 25 μL of L-tyrosine (0.3 mg/mL) was added and the absorbance was measured at 510 nm (kinetic model, every 5 min). Next, two time points (t_1_ and t_2_) were selected in the linear range of the graph. All samples were tested in triplicate. Kojic acid was used as a standard. The tyrosinase inhibition was calculated using the following equation:

$${INH}_{tyrosinase}=(\frac{\Delta A_{C}-{\Delta A}_{S}}{{\Delta A}_{C}})\times 100\%$$

A_S_—the difference in absorbance between time t_2_ and t_1_ for sample

A_C_—the difference in absorbance between time t_2_ and t_1_ for positive control

### 3.5. Chemical Panel

#### 3.5.1. Total Polyphenols Content (TPC)

To each well, 25 μL of extract (1.0 mg/mL), 25 μL of 9-fold diluted FC reagent, and 200 μL of distilled water were added. The plate was incubated for 5 min, and then, 25 μL saturated Na_2_CO_3_ solution was added. The plate thus prepared was incubated for 60 min in the dark at room temperature. The absorbance of the analyzed samples was measured at λ = 750 nm. Each sample was performed in triplicate. The results were expressed as milligrams of gallic acid equivalents (GAEs) per gram of sample [mg GAE/g], with the calibration curve shown in Supplementary Figure S5 [49].

#### 3.5.2. Total Flavonoids Content (TFC)

To each well was added 25 μL of extract (1.0 mg/mL), 75 μL of ethanol, 10 μL of aluminum trichloride solution, 10 μL of potassium acetate solution, and 130 μL of distilled water. The plate thus prepared was incubated for 30 min in the dark at room temperature. Absorbance was measured at λ = 510 nm. Each sample was performed in triplicate. The results were expressed as milligrams of quercetin equivalents (QEs) per gram of sample [mg QE/g], with the calibration curve shown in Supplementary Figure S6 [50].

#### 3.5.3. Total Phenolic Acids Content (TPAC)

To each 96-well was added 25 μL of extract (1.0 mg/mL), 150 μL distilled water, 25 μL solution of HCL, 25 μL of Arnov’s reagent and 25 μL of NaOH solution. The absorbance of the analyzed samples was measured at λ = 492 nm. The results were expressed as milligrams of caffeic acid equivalents (CAEs) per gram of sample [mg CAE/g] with the calibration curve shown in Supplementary Figure S7 [51].

#### 3.5.4. Chromatographic Analysis

All reagents, including the standards, formic acid, and MS-grade acetonitrile, were sourced from Sigma-Aldrich (St. Louis, MO, USA). The mass spectrometry data were recorded using an Infinity Series II ultra-high-performance liquid chromatography (UHPLC) system coupled with an Agilent 6224 ESI/TOF mass spectrometer (Agilent Technologies, Santa Clara, CA, USA). The chromatographic conditions were set as follows: an RP18 reversed-phase Titan column (Supelco, Sigma-Aldrich, Burlington, MA, USA), with dimensions of 10 cm × 2.1 mm i.d. and a particle size of 1.9 µm. The column temperature was maintained at 30 °C, with a mobile phase flow rate of 0.2 mL/min. The mobile phases used were as follows: solvent A, which consisted of water with 0.05% formic acid, and solvent B, which contained acetonitrile with 0.05% formic acid. The gradient elution profile was as follows: 0–8 min from 97% A to 95% A, 8–15 min held at 95% A, 15–29 min from 95% A to 85% A, 29–40 min held at 85% A, 40–50 min from 85% A to 80% A, and 50–60 min from 80% A to 65%.

LC-MS parameters included a drying gas temperature of 325 °C, a drying gas flow rate of 8 L/min, a nebulizer pressure of 30 psi, a capillary voltage set to 3500 V, and a 65 V skimmer. The fragmentor voltage was set to 220 V. Data acquisition occurred over a mass-to-charge ratio range of 100 to 1200 m/z in negative ion mode.

For quantitative analysis, a Merck EliteLaChrom chromatograph equipped with a PDA detector and EZChrom Elite software (Version 3.3.2 SP2 build 3.3.2.1037) was utilized (Merck, Darmstadt, Germany), with an RP18 Kinetex column (Phenomenex, Torrance, CA, USA), dimensions of 25 cm × 4.6 mm i.d. and a particle size of 5 μm. The flow rate was adjusted to 1.0 mL/min, and spectral data were collected between 190 and 400 nm. To confirm compound identities, retention times and UV spectra were compared to those of standard substances. Quantitative measurements were conducted at specific wavelengths: 260 nm for protocatechuic acid, 325 nm for chlorogenic acid, and 325 nm for dicaffeoylquinic acid.

### 3.6. Cytotoxicty Panel

#### Cell Culture and Cytotoxicity Assessment

A375 (ATCC:CRL-1619) human melanoma cell line was maintained in Dulbecco’s Modified Eagle’s Medium (DMEM) with fetal bovine serum (FBS) at a final concentration of 10%. UACC-647 (CVCL_4049) and SK-MEL-30 (DSMZ:ACC 151) melanoma cells were cultured in 90% RPMI-1640 medium supplemented with 10% FBS. BJ foreskin fibroblasts (ATCC:CRL-2522) served as control. Eagle’s Minimum Essential Medium (EMEM) with 10% FBS was used to support the growth of BJ cells.

All cell lines were routinely incubated at 37 °C with 5% CO_2_ on tissue culture-treated Petri dishes. The cells were expanded for a few passages and seeded onto 96-well plates. The seeding density for SK-MEL-30 and UACC-647 cell lines was 10^4^ cells/well. A375 and BJ were used at 12 × 10^3^ and 8 × 10^3^ cells/well, respectively. The cells were allowed to attach overnight. The next day, the cells were exposed to *E. senticosus* fractionated extracts at decreasing concentrations starting from 200 µg/mL (two-fold dilution, eight doses) or vehicle (DMSO, 0.1%). Alternatively, the cells were treated with doxorubicin (DOX) to determine the inhibitory concentrations causing the 10, 50, and 90% of maximal inhibition (IC_10_, IC_50_, and IC_90_, respectively) of cellular viability, independently for each melanoma cell line. Then, the melanoma cells were treated with the DOX at IC_10_, IC_50_, or IC_90_ followed by the addition of the *E. senticosus* fractionated extracts at the dose of 200 µg/mL. Upon 24 h incubation, the viability of the cells was assessed by MTT assay. Recorded absorbance (Abs for λ = 570 nm) values for all samples were reduced by the average value for blank wells, normalized to the average absorbance for vehicle controls, and expressed as percentage, as indicated by the following equation:

$$Percentage\;Viability\;\left(\%\right)=\frac{{Abs}_{sample}-\bar{{Abs}_{blank}}}{\bar{{Abs}_{vehicle\_control}}-\bar{{Abs}_{blank}}}\times 100$$

### 3.7. Statistical Analysis

Dose–response relationships were analyzed using GraphPad Prism (v8.4.3) installed on a standard workstation. Data were fitted using a nonlinear regression model with a sigmoidal function to estimate the IC_50_ values. The IC_10_ and IC_90_ parameters were obtained via an online tool available at https://www.graphpad.com/ (accessed on 20 May 2023).

All statistical procedures, including standard deviation calculations, one-way ANOVA, Tukey’s HSD test, PCA, and correlation analysis were conducted using Statistica software (version 13.1 PL, StatSoft, Kraków, Poland) for Windows. The threshold for statistical significance was set at α = 0.05.

## 4. Conclusions

*Eleutherococcus senticosus* is a plant that has been used in traditional medicine for centuries. Studies on the methanol extract from the root of ES and its fractions have shown broad biological activity, indicating its therapeutic potential in the treatment of lifestyle diseases such as diabetes, inflammation, and Alzheimer’s disease. The extract and its fractions exhibit strong anti-inflammatory, anti-neurodegeneration, metabolic, and anti-aging effects. The EtOAc and BuOH fractions demonstrated the highest anti-inflammatory activity. The extract also has strong antioxidant properties due to its high content of chlorogenic acid.

Our study demonstrated that fractions from *E. senticosus* root are not toxic to normal fibroblasts (BJ) or melanoma cell lines (SK-MEL-30, UACC-647, A375). However, when administered simultaneously with doxorubicin (DOX), a clear protective effect of the EtOAc and BuOH fractions was observed, as they attenuated the cytotoxic activity of the drug against melanoma cells. This phenomenon may have significant clinical implications, as it suggests that the concurrent use of *E. senticosus* preparations during chemotherapy could potentially reduce the effectiveness of anticancer treatment. These findings highlight the need for further research on interactions between adaptogens and cytotoxic drugs, particularly in the context of oncology.

In conclusion, this research confirmed the rightness of the ethnopharmacological use of *E. senticosus* in the prevention and treatment of lifestyle-related diseases. This research means that there are molecular mechanisms that can be stimulated by ES. Nevertheless, further studies are required to confirm the health-promoting properties of *E. senticosus* in animal models.

## Figures and Tables

**Table 1:** Antioxidants’ activity of *Eleutherococcus senticosus* fractions. IC_50_ values are shown in µg/mL. Different superscript lowercase letters indicate a statistically significant difference between the fractions themselves and between the fractions and control within the same column, with *p* < 0.05.

Fractions	DPPH	ABTS	O_2_^•−^
HEX	388.07 ± 26.36 ^a^	132.67 ± 1.45 ^a^	NA
EtOAc	82.73 ± 0.065 ^b^	9.92 ± 0.17 ^b^	202.16 ± 5.71 ^a^
BuOH	120.57 ± 12.15 ^c^	23.25 ± 0.24 ^c^	153.54 ± 2.95 ^a^
H_2_O	133.68 ± 0.89 ^c^	22.73 ± 0.05 ^c^	NA
BHA	62.52 ± 4.13 ^d^	2.35 ± 0.11 ^d^	
AA	24.93 ± 0.28 ^e^	2.27 ± 0.07 ^d^	44.77 ± 0.91 ^b^
TROLOX	13.68 ± 3.53 ^e^	2.85 ± 0.18 ^d^	

HEX—hexane, EtOAc—ethyl acetate, BuOH—butanol, H_2_O—water.

**Table 2:** Anti-enzymatic activity of fractions. IC_50_ values are shown in µg/mL. Different superscript lowercase letters indicate a statistically significant difference between the different acids within the same column, with *p* < 0.05.

Fractions	Anti-Inflammatory	Anti-Neurodegenerative		Metabolic	Anti-Aging
	Hyal	AChE	BuChE	Amyl	Tyr
HEX	NA	280.00 ± 49.72 ^a^	245.72 ± 11.82 ^a^	97.34 ± 9.18 ^a^	324.80 ± 30.73 ^a^
EtOAc	125.24 ± 12.29 ^a^	NA	301.23 ± 2.54 ^a^	281.63 ± 7.77 ^b^	147.12 ± 8.28 ^b^
BuOH	382.04 ± 33.01 ^b^	1000>	1000>	NA	214.51 ± 37.91 ^c^
H_2_O	361.41 ± 43.57 ^b^	NA	NA	NA	230.45 ± 3.78 ^c^
Escin	390.8 ± 0.81 ^b^				
Kojic acid					34.33 ± 1.22 ^d^
Donepezil		22.68 ± 0.67 ^b^	87.17 ± 1.92 ^b^		
Acarbose				384.34 ± 27.84 ^c^	

HEX—hexane, EtOAc—ethyl acetate, BuOH—butanol, H_2_O—water.

**Table 3:** Chemical composition of fractions from the *Eleutherococcus senticosus* roots. Different superscript lowercase letters indicate a statistically significant difference between the different acids within the same column, with *p* < 0.05.

Fractions	TPC [mgGAE/g]	TFC [mgQE/g]	TPAC [mgCAE/g]	Mass of Fraction [g]
HEX	2.88 ± 0.30 ^a^	6.37 ± 0.39 ^a^	1.21 ± 0.14 ^b^	0.47
EtOAc	26.77 ± 1.10 ^b^	21.35 ± 3.34 ^b^	3.13 ± 0.54 ^a^	1.34
BuOH	7.33 ± 0.14 ^c^	3.90 ± 1.03 ^a^	0.43 ± 0.06 ^b^	9.89
H_2_O	4.52 ± 0.62 ^a^	5.25 ± 2.06 ^a^	0.67 ± 0.048 ^b^	4.81
Σ	41.50	36.87	5.44	

HEX—hexane, EtOAc—ethyl acetate, BuOH—butanol, H_2_O—water.

**Table 4:** Content of phenolic acids and eleutheroside B in fractions obtained from *E. senticosus* roots.

	PA	CA	ChA	4,5-DCA	3,5-DCA	Eleuth B
	Mean ± SD	Mean ± SD	Mean ± SD	Mean ± SD	Mean ± SD	Mean ± SD
HEX	0.06 ± 0.000	0.12 ± 0.00	6.38 ± 0.00	0.20 ± 0.00	0.88 ± 0.01	0.13 ± 0.01
EtOAc	8.02 ± 0.01	9.96 ± 0.05	117.57 ± 0.87	16.58 ± 0.71	103.36 ± 0.39	1.13 ± 0.10
BuOH	<LOD	<LOQ	48.06 ± 0.23	0.12 ± 0.03	0.53 ± 0.07	2.49 ± 0.20
H_2_O	<LOD	<LOQ	65.56 ± 0.77	<LOD	0.12 ± 0.01	0.69 ± 0.06

PA—protocatechic acid, CA—caffeic acid, ChA—chlorogenic acid, DCA—dicaffeoylquinic acid, Eleuth B—eleutheroside B, HEX—hexane, EtOAc—ethyl acetate, BuOH—butanol, H_2_O—water.

**Table 5:** UHPLC-DAD/ESI-TOF-MS analysis of phenolic composition of the ethyl acetate fraction.

	Rt (min)	Observed Ion Mass [M-H]-/(Fragments)	Δ ppm	Formula	Identified	(mg/g)
1	5.57	153.01973	2.58	C_7_H_6_O_4_	protocatechuic acid	7.68 ± 0.18
2	8.73	137.02481	2.84	C_7_H_6_O_3_	hydroxybenzoic acid	+
3	15.20	179.03505 (135,191)	0.38	C_9_H_8_O_4_	caffeic acid	8.64 ± 0.31
4	16.42	353.08835 (191,179)	1.54	C_16_H_18_O_9_	chlorogenic acid	118.5 ± 6.2
5	18.40	353.08843 (191,179)	1.76	C_16_H_18_O_9_	cryptochlorogenic acid	6.81 ± 1.01
6	23.20	337.09312	0.68	C_16_H_18_O_8_	*p*-coumaroylquinic acid	1.78 ± 0.10
7	25.37	367.10366	0.55	C_17_H_20_O_9_	feruloylquinic acid	2.61 ± 0.20
8	36.84	515.12021 (353)	1.38	C_25_H_24_O_12_	3,5-dicaffeoylquinic acid	75.1 + 4.2
9	37.63	515.12048 (353)	1.90	C_25_H_24_O_12_	dicaffeoylquinic acid	93.3 + 3.3
10	50.90	515.12035 (353)	1.65	C_25_H_24_O_12_	4,5-dicaffeoylquinic acid	7.11 ± 0.23
11	59.33	577.13521 (193, 385)	0.10	C_30_H_26_O_12_	diferulic acid derivative	3.32 ± 0.21

## Data Availability

The data presented in this study are available on request from the corresponding author.

## Supplementary Materials

The following supporting information can be downloaded at: https://www.mdpi.com/article/10.3390/ijms26125619/s1.
