# Supplementary material for: Chemical Composition and In Vitro Biological Activity of the Polar and Non-Polar Fractions Obtained from the Roots of Eleutherococcus senticosus (Rupr. et Maxim.) Maxim
Source: Int J Mol Sci. 2025 Jun 12;26(12):5619. doi: 10.3390/ijms26125619 (PMC12192905; doi:10.3390/ijms26125619)
Supplement: Supplementary file 1 [file ijms-26-05619-s001.zip › ijms-3631326-supplementary.pdf]

Supplementary material:

a

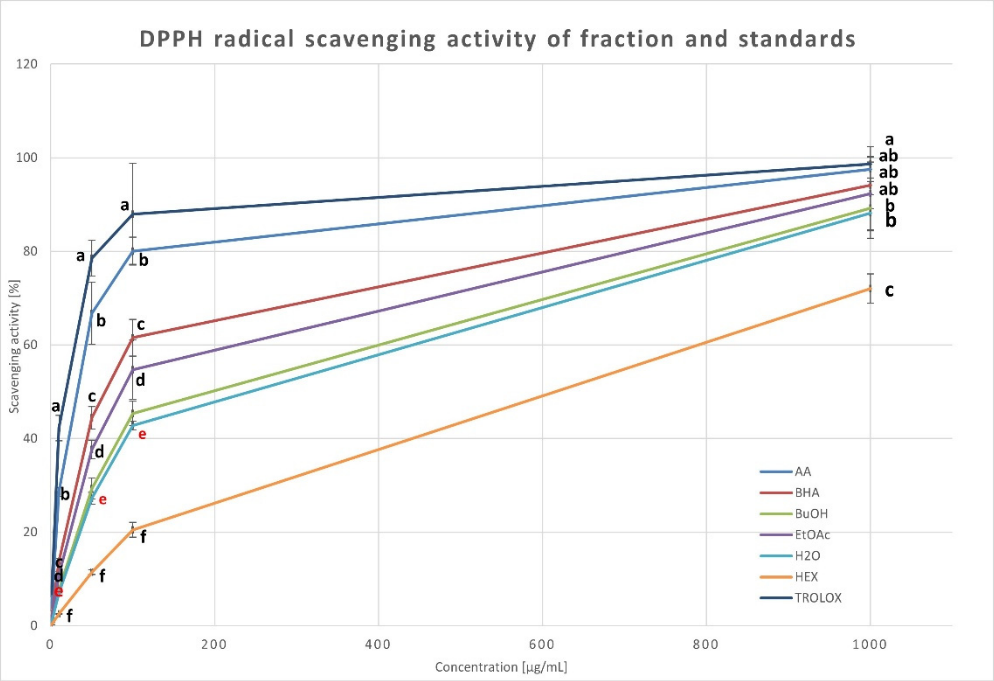

b

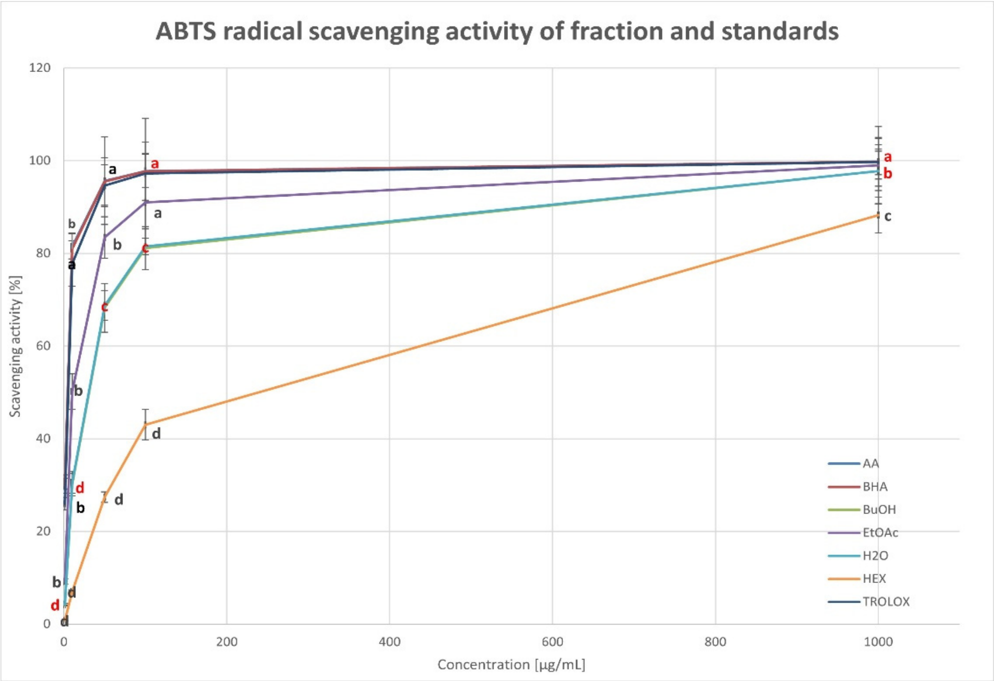

c

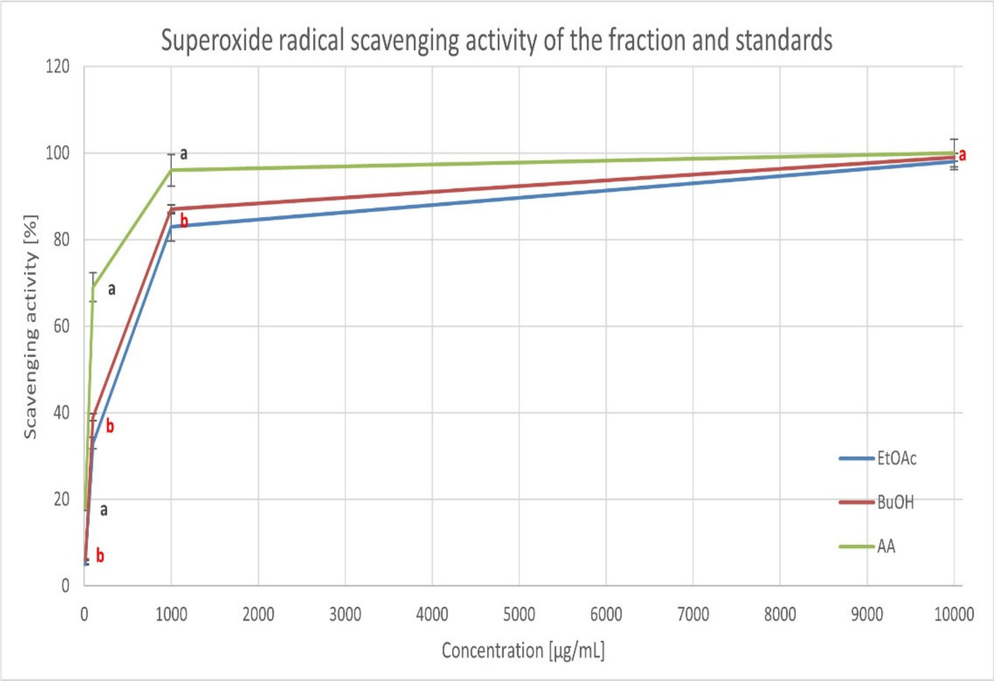

Figure S1. DPPH (a), ABTS (b) superoxide (c) radical scavenging activity (%) of extract fractions and antioxidant standards (Trolox, ascorbic acid, BHA) depending on concentration (1000 - 1 µg/mL). Different superscript lowercase letters indicate a statistically significant difference between the fractions themselves and between the fractions and control within the same column, with $p < 0.05$. For DPPH at 1 µg/mL, ascorbic acid (AA), Trolox, BHA, ethyl acetate (EtOAc), and butanol (BuOH) fractions showed no significant differences among each other, while water (H_2O) and hexane (HEX) fractions were significantly different from these but not significantly different from each other. In the DPPH and ABTS assays, no significant difference was observed between the BuOH and H_2O fractions, whereas in the superoxide assay, no significant difference was observed between the BuOH and EtOAc fractions.

a

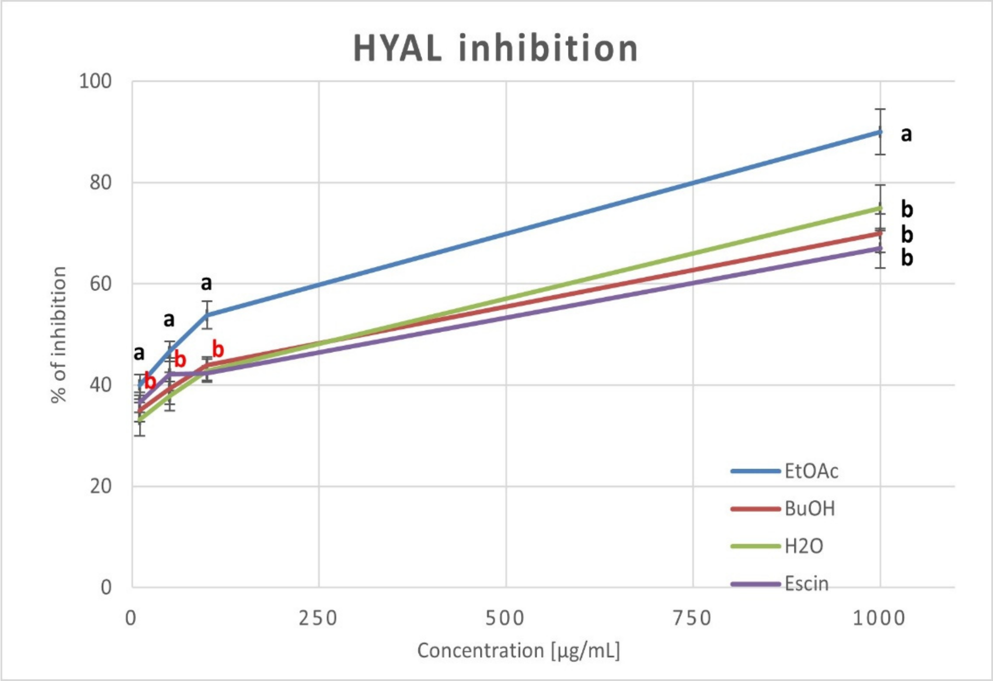

b

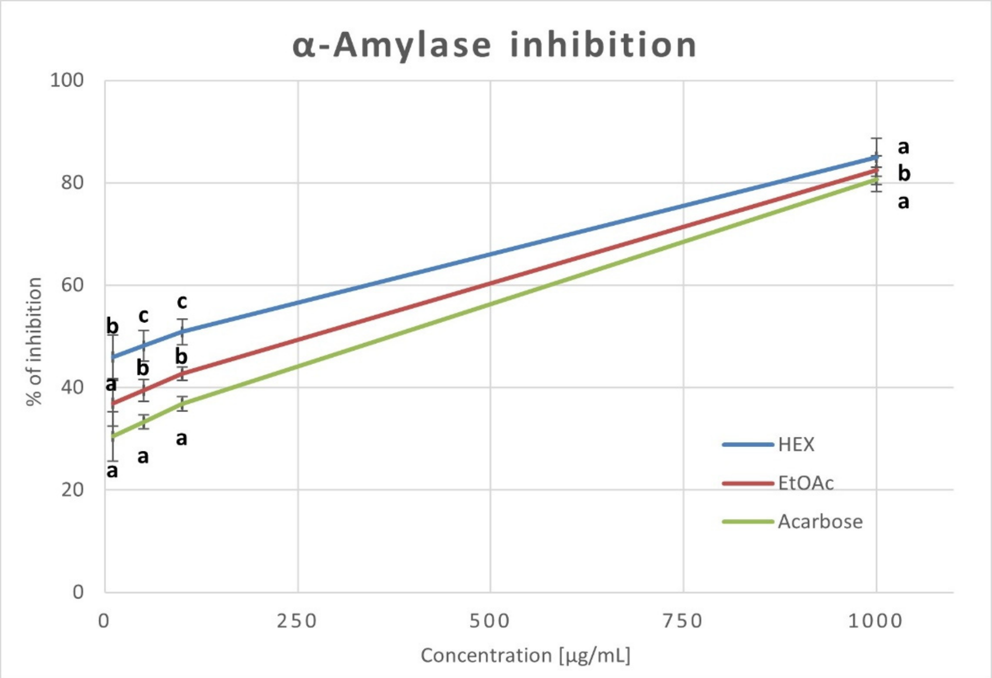

α-Amylase inhibition
% of inhibition
Concentration [µg/mL]
HEX
EtOAc
Acarbose

c

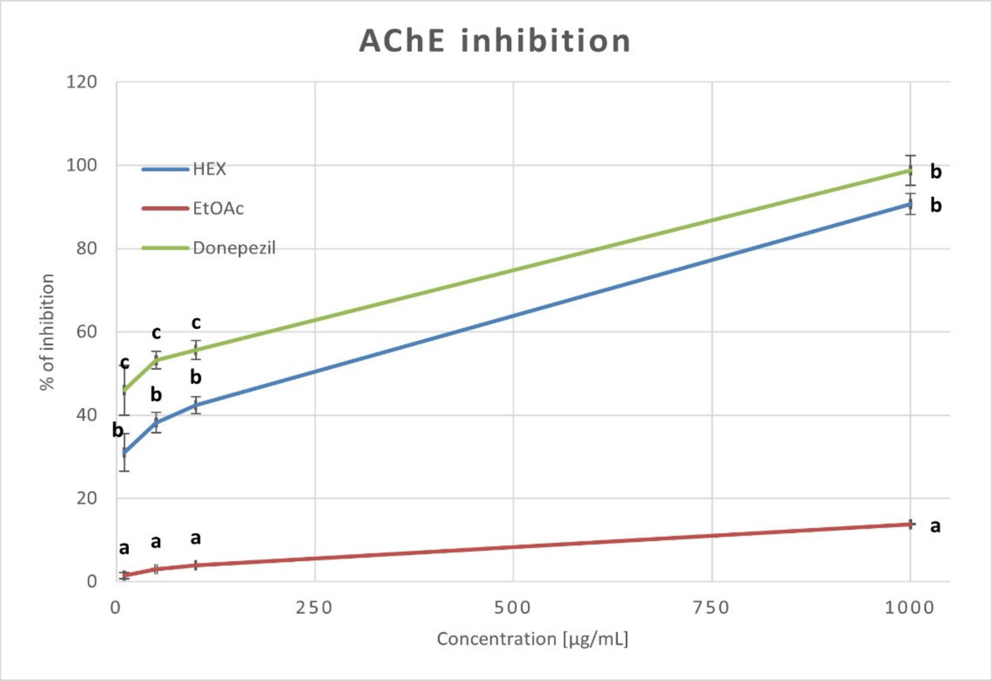

AChE inhibition
% of inhibition
Concentration [µg/mL]
HEX
EtOAc
Donepezil

d

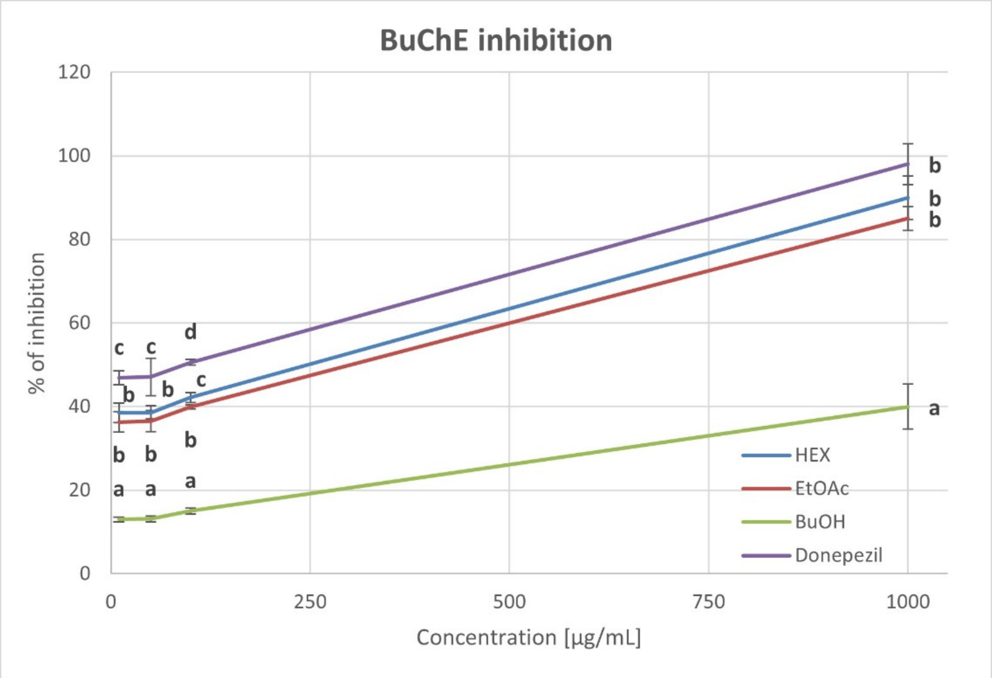

e

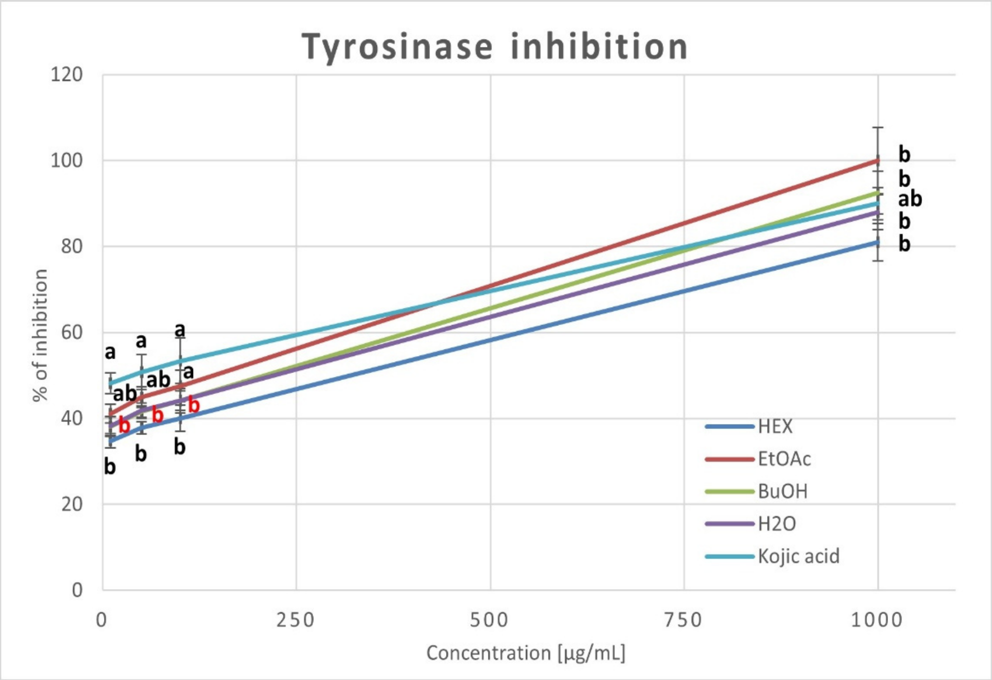

Figure S2. Anti-enzymatic activity of plant extracts at different concentrations (1000-10 µg/mL). Statistical differences are indicated by lowercase letters, and standard deviation values are represented by vertical bars. Red letters on the hyaluronidase chart indicate a shared statistical group for the BuOH and H_2O fractions as well as escin, while on the tyrosinase chart they indicate a shared statistical group for the BuOH and H_2O fractions. a – for hyaluronidase b – for α-amylase c – for acetylcholinesterase d – for butyrylcholinesterase e – for tyrosinase

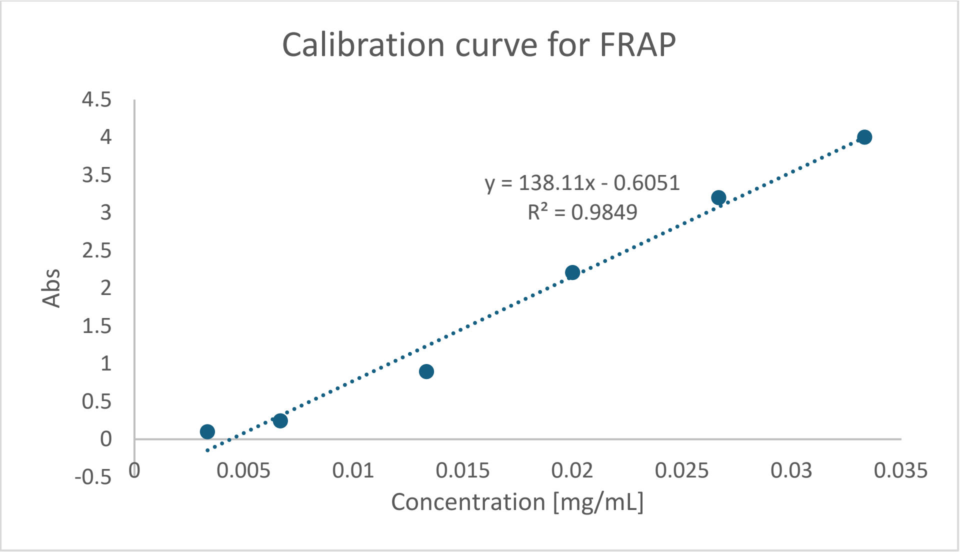

Figure S3. Calibration curve for Ferric Ion Reducing Antioxidant Power Assay.

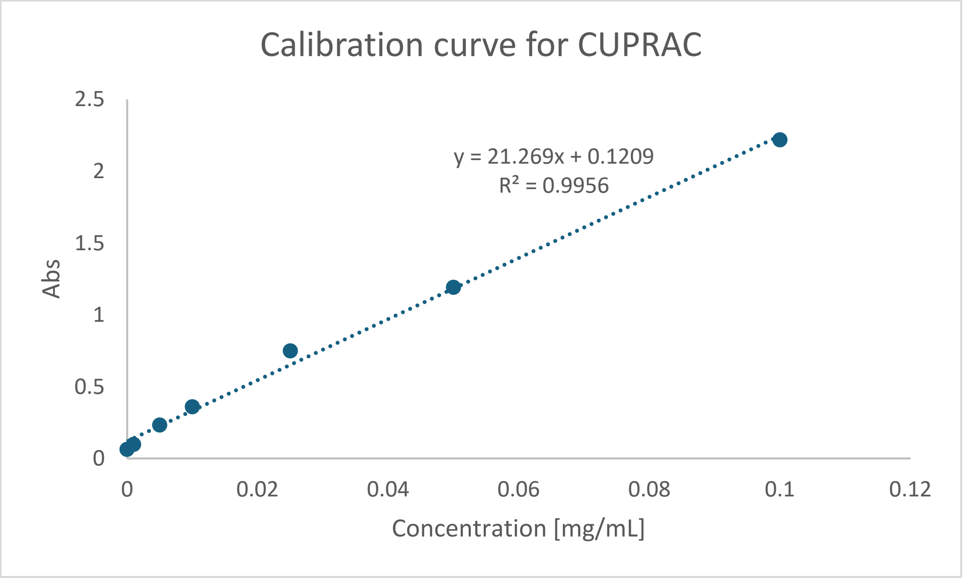

Figure S4. Calibration curve for Cupric Ion Reducing Antioxidant Capacity Assay.

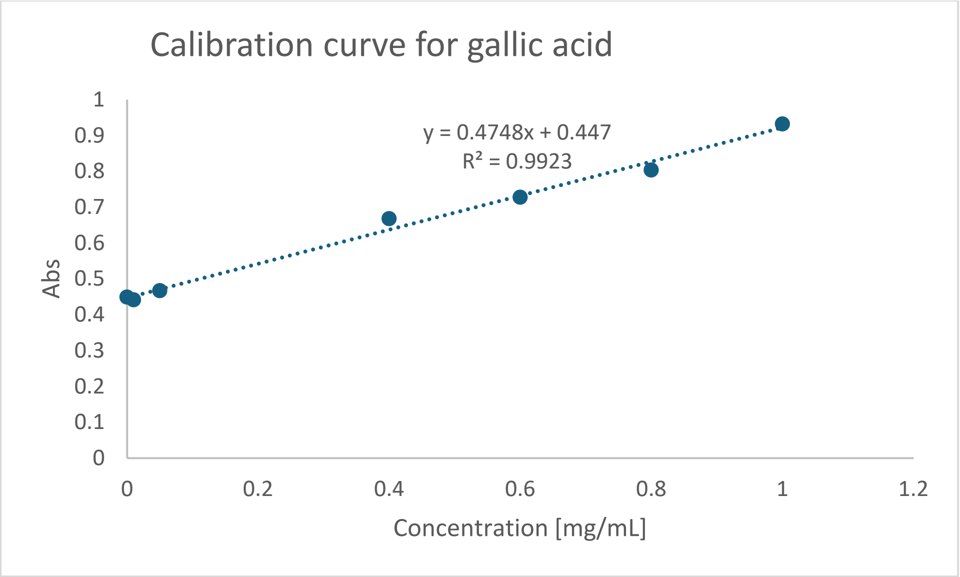

Figure S5. Calibration curve for gallic acid.

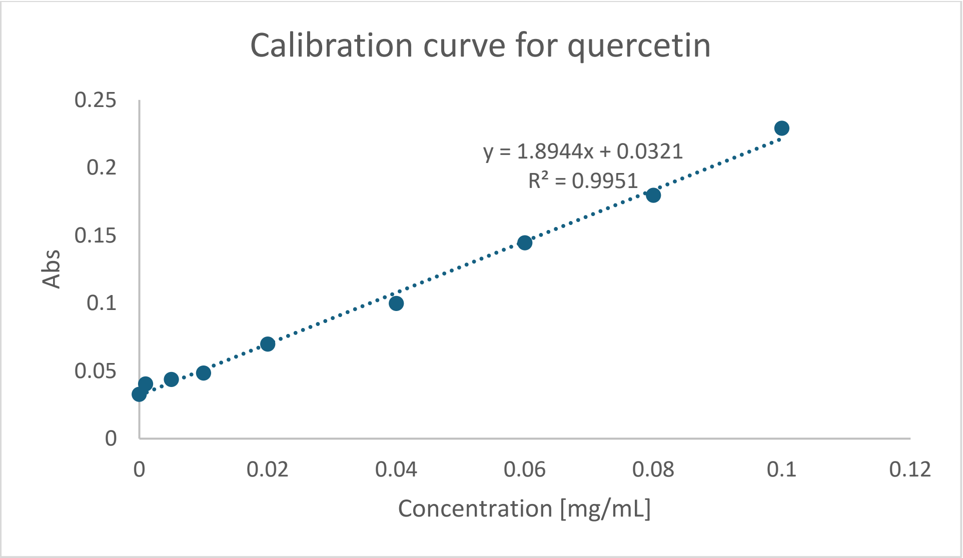

Figure S6. Calibration curve for quercetin.

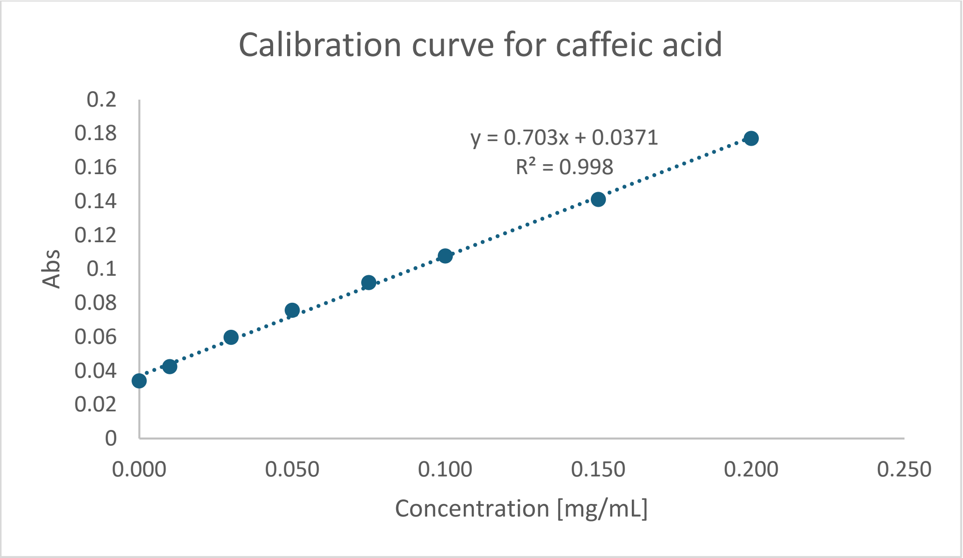

Figure S7. Calibration curve for caffeic acid.
